# Non-Cutaneous Visceral Kaposi's Sarcoma Diagnosis Confounded by Mycobacterium Avium Complex Lymphadenitis

**DOI:** 10.7759/cureus.36765

**Published:** 2023-03-27

**Authors:** Andrew B Herson, John M Sousou, Kimberly Boldig, Falguni Patel, Pramod Reddy

**Affiliations:** 1 Internal Medicine, Lake Erie College of Osteopathic Medicine, Bradenton, USA; 2 Internal Medicine, University of Florida College of Medicine, Jacksonville, USA

**Keywords:** mycobacterium avium, kaposi's sarcoma, aids-defining illness, people living with hiv/aids, lymphadenitis, mycobacterium avium-complex, visceral kaposi sarcoma

## Abstract

Kaposi's sarcoma (KS) is an Acquired Immune Deficiency Syndrome (AIDS)-defining illness, with cutaneous KS being a more common presentation. Visceral involvement, particularly in the gastrointestinal (GI) tract, without cutaneous involvement, is rare. Consisting of generally non-specific symptoms, GI-KS can have potentially fatal outcomes, including hemorrhage or perforation, making prompt diagnosis and treatment imperative. Our case describes a 31-year-old male with AIDS who presented with a neck mass and purulent, bloody rectal drainage. The neck mass was biopsied and identified as caseated necrotic cervical lymphadenitis caused by Mycobacterium avium complex (MAC). The patient presented with rectal drainage, and additional abdominal necrotic lymph nodes were discovered on CT. A subsequent colonoscopy was completed, confirming the diagnosis of visceral KS. Delayed diagnosis of visceral KS can lead to an extensive, widespread disease requiring adjuvant and prolonged treatment. Prompt diagnosis can reduce morbidity and mortality. This case aims to shed light on a rare presentation of a common disease state with potentially fatal complications and emphasizes the importance of maintaining a broad differential diagnosis.

## Introduction

Kaposi's sarcoma (KS) is an aggressive vascular tumor caused by human herpesvirus 8 (HHV-8) that can present with cutaneous or visceral manifestations. KS is typically known as an AIDS-defining malignancy, and it is the most common AIDS-associated tumor [1]. KS affects HIV patients at a rate up to 1000 times greater than the general population [2]. The distribution of HHV-8 is not uniform, and its occurrence differs significantly across specific geographic areas. The greatest prevalence is observed in Sub-Saharan Africa, with rates as high as 50% in Uganda and 30.7% in Cape Town, South Africa. The Mediterranean region has rates ranging from 10% to 30%. In contrast, the general population in Europe, Asia, and the United States has a prevalence of less than 5% [3]. Cutaneous KS is the most common manifestation of this neoplasm and typically affects the skin and oropharynx. Less commonly, cases of gastrointestinal (GI) and pulmonary organ involvement occur that can lead to severe disease progression and a poor overall prognosis. KS may also present as an immune reconstitution inflammatory syndrome (KS-IRIS) or inflammatory cytokine syndrome (KICS), both of which can cause rapid clinical deterioration and death [4].

Visceral organ involvement of KS without the presence of cutaneous disease is a rare occurrence that is highly aggressive and fatal if treatment is delayed. The most common sites of involvement of visceral KS in the GI tract are the stomach, colon, and small intestine; virtually any organ from the mouth to the anus may be affected [1]. Identifying cases of gastrointestinal KS can be particularly challenging since the involvement of the gastrointestinal (GI) tract usually does not cause any symptoms. When symptoms do occur, they are often non-specific and can include nausea, vomiting, abdominal pain, and diarrhea. More severe complications that have been previously reported include perforation, obstruction, and hemorrhage [1]. We present the case of a patient with visceral KS whose diagnosis was confounded by concurrent mycobacterial avium complex (MAC) lymphadenitis.

## Case presentation

A 31-year-old male with a past medical history of AIDS (clusters of differentiation 4 (CD4) count of 194; compliant with bictegravir, emtricitabine, and tenofovir alafenamide therapy) and intravenous drug use presented to our institution with a right-sided neck mass. He reported noticing the mass one month prior to the presentation. He described associated pain and decreased range of motion. He also admitted to having purulent stools and rectal bleeding. He reported anoreceptive intercourse and constipation.

Initial vital signs on presentation were as follows: blood pressure of 100/62, pulse rate of 98 bpm, temperature of 99.3°F, respiratory rate of 14 breaths per minute, and 100% oxygenation on room air. Laboratory tests included a chemistry panel that was within normal limits. A complete blood count showed normocytic anemia (Table 1). The white blood cell count differential showed elevated neutrophil percentage.

**Table 1:** Laboratory values

	Reference range	Laboratory value
White blood cells (WBC)	4.5-11 thou/mm^3^	9.22 thou/mm^3^
Hemoglobin (Hb)	14-18 g/dL	10.3 g/dL
Hematocrit (Hct)	40-54 %	31.7 %
Mean corpuscular volume (MCV)	82-101 fL	83.2 fL
Platelets	140-440 10^9^/L	297 10^9^/L
Neutrophils %	34-73 %	79.5%
Lymphocytes %	25-45 %	13%
Ferritin	30-400 ng/mL	169 ng/mL
Iron	32-159 µg/dL	10 µg/dL
Total iron binding capacity (TIBC)	261-390 mcg/dL	232 mcg/dL
Transferrin	200-360 mg/dL	183 mg/dL

**Figure 1:**
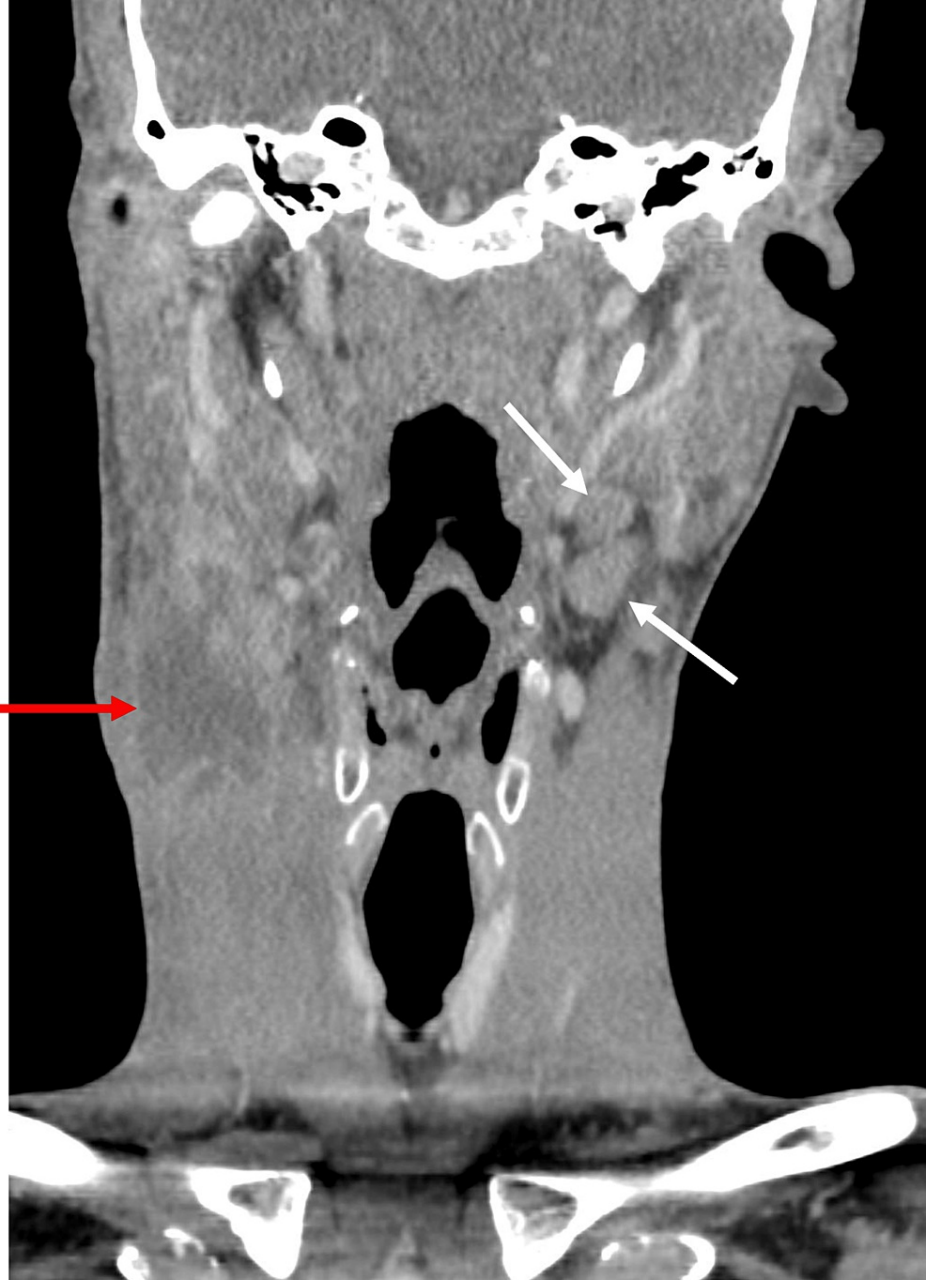
Coronal reformat CT of the neck (soft tissue) CT of the neck (soft tissue) with contrast coronal reformat demonstrated a large conglomerate right level 2A/3 lymph node with internal areas of non-enhancement (red arrow). There are additional enlarged left cervical lymph nodes (white arrows).

The patient was found positive for Mycoplasma genitalium and completed treatment. Further sexually transmitted disease (STD) testing was negative. A computed tomography (CT) of the neck soft tissues with intravenous contrast (IV) demonstrated bilateral cervical lymphadenopathy with areas of internal necrosis and caseation. On the right side, a lymph node conglomerate measured up to 7.3 centimeters (Figure 1). On the left side, a 4.2 cm nodal conglomerate was identified. The patient was placed in isolation due to concern for tuberculosis infection. An incision and drainage procedure of the neck mass was performed. An acid-fast bacilli (AFB) smear showed 4+ AFB, raising further concern for tuberculosis lymphadenitis. QuantiFERON later returned negative results, and AFB cultures speciated into Mycobacterium avium complex (MAC). Mycobacterium tuberculosis (M. tuberculosis) polymerase chain reaction (MTB PCR) was negative for M. tuberculosis complex DNA. The patient was started on azithromycin, ethambutol, and moxifloxacin for one year for MAC treatment.

**Figure 2:**
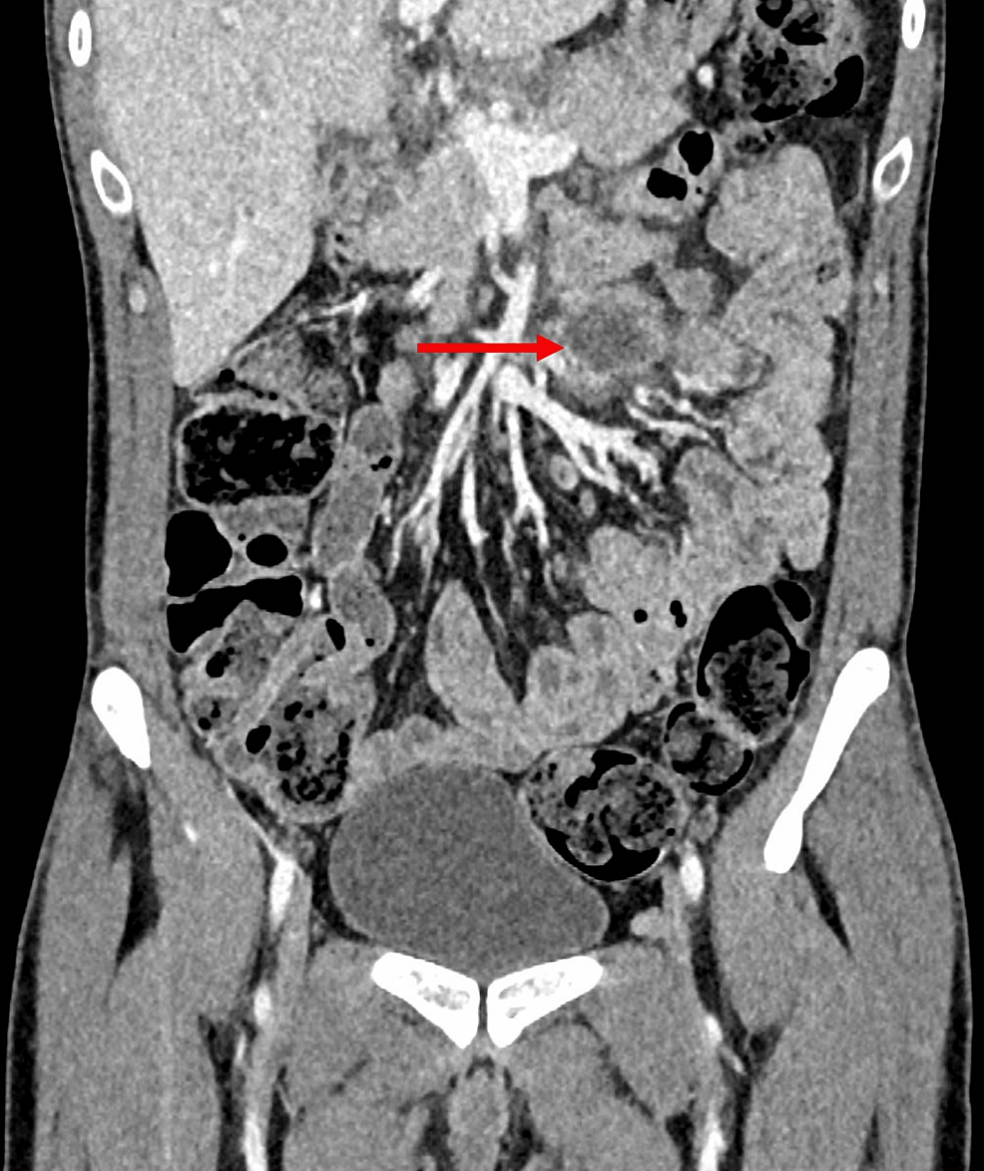
Coronal reformat CT of the abdomen and pelvis A CT of the abdomen and pelvis with intravenous (IV) contrast coronal reformat demonstrating an enlarged conglomerate mesenteric lymph node with internal areas of non-enhancement centered in the left hemiabdomen (red arrow).

A CT of the abdomen and pelvis was ordered due to the patient’s complaint of rectal bleeding and to assess for further lymphadenopathy (Figure 2). The exam showed extensive necrotic lymphadenopathy within the abdomen, scattered hepatic hypodensities, circumferentially enhanced rectal wall thickening, and circumferentially enhanced esophageal thickening.

Gastroenterology (GI) was consulted due to rectal bleeding and a possible colonoscopy. The GI team was unable to perform a colonoscopy as the patient was unwilling to complete the preparation. General surgery performed an inguinal lymph node excision and biopsy. Pathology later demonstrated a spindle-cell neoplasm consistent with Kaposi's sarcoma. Histologic sections showed areas of subcapsular spindle cell proliferation with HHV8+ and CD34 variables.

**Figure 3:**
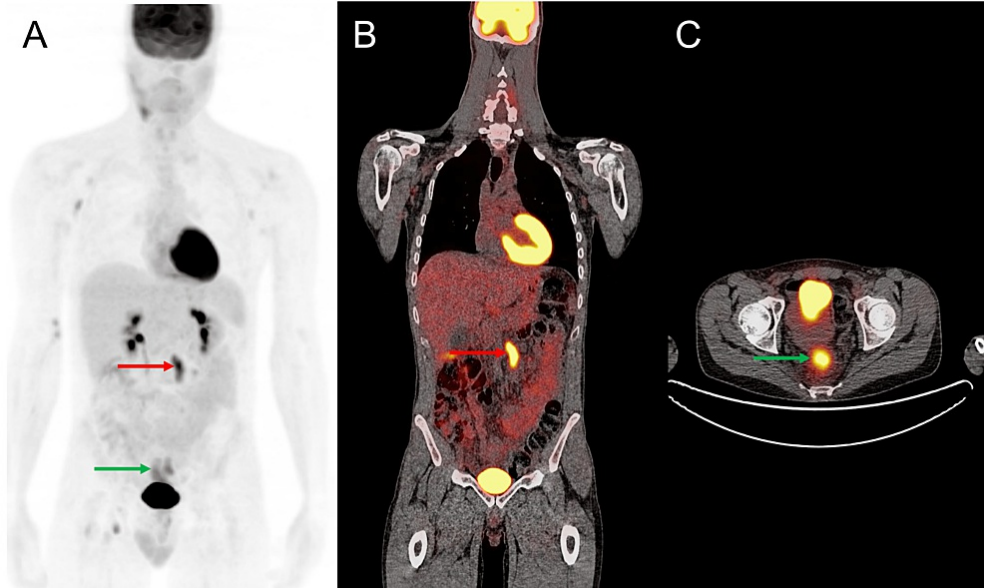
PET-CT images Maximum intensity projection (MIP) image of PET-CT (A) coronal-fused PET-CT (B) axial-fused PET-CT (C) demonstrating an F-fluorodeoxyglucose (FDG)-avid enlarged mesenteric lymph node with a maximum standardized uptake value (SUV) of 9.26 (red arrows). Additionally, there are FDG-avid rectal nodules with a maximum SUV of 16.33 (green arrows).

The patient left the hospital against medical advice. He had an inconsistent outpatient follow-up but was able to make it to an oncology appointment and complete a positron emission tomography CT (PET CT) (Figure 3). The PET CT demonstrated multiple index hypermetabolic lesions within the distal rectum. Additionally, hypermetabolic lymph nodes within the right cervical, bilateral axillary, right subpectoral, and bilateral superficial inguinal regions were seen

The patient returned to the emergency department approximately two months later with worsening abdominal pain. A CT of the abdomen and pelvis showed worsening bowel wall thickening and inflammatory changes in the rectosigmoid colon, extensive mesenteric and retroperitoneal lymphadenopathy, and necrotic conglomerate lymphadenopathy in the left upper abdomen mesentery. The GI team performed a colonoscopy and found a mass lesion and ulceration 3 cm from the anal verge. Pathology results confirmed Kaposi's sarcoma. Oncology diagnosed Kaposi's sarcoma with cT1 (visceral involvement), I1 (CD4150), and S1 (recent Mycobacterium avium complex (MAC) infection), which correlated to poor-risk disease. He was started on liposomal doxorubicin (20 mg/m2) every three weeks. The patient has remained compliant with anti-retroviral therapy (ART) therapy and has tolerated chemotherapy; further follow-up is scheduled.

## Discussion

A thorough evaluation of the lymphadenopathy in our patient allowed for a concurrent diagnosis of MAC and Kaposi's sarcoma. MAC is an acid-fast, slow-growing bacterium that is typically acquired through exposure to contaminated soil and water. Its ability to spread through water is due to its resistance to chlorine and biocides [5]. The incidence of MAC infections is increasing at a rate of 5%-10% annually [5]. In individuals with AIDS, MAC infections typically occur when the CD4 count drops below 50 cells/µl. The infection often presents as a disseminated illness, causing symptoms such as fever, fatigue, weight loss, abdominal pain, and an enlarged liver and spleen. Approximately 40% of patients may also experience diarrhea, as the gastrointestinal tract is typically the initial site of infection [6].

MAC infections can present as pulmonary disease, disseminated disease, or lymphadenitis. MAC lymphadenitis typically affects the cervical chain and most often impacts children [7]. It can also occur in patients undergoing anti-retroviral therapy (ART) in the form of immune reconstitution inflammatory syndrome (IRIS). IRIS is an inflammatory reaction that occurs in response to an increase in CD4 T-cell counts following the initiation or alteration of ART therapy [8]. IRIS symptoms associated with MAC infection may include fever, fatigue, and inflammation of the lymph nodes (lymphadenitis) [8]. Treatment options for MAC lymphadenitis may include surgical excision or a combination of antimicrobial medications, such as a macrolide, ethambutol, and/or rifampin [7].

KS is a tumor caused by the growth of infected lymphatic endothelial cells in patients with HHV-8 [1]. KS is classified into several types, including classic, iatrogenic, endemic, and AIDS-associated, with the latter being the most prevalent [9]. The presentation may range from mild skin changes to widespread illness affecting internal organs. Nearly 100% of all types of Kaposi's sarcoma, whether related to HIV or not, contain Kaposi's sarcoma herpes virus (KSHV) viral DNA and express at least one viral protein, called the latency-associated nuclear antigen (LANA), along with all the viral microRNAs [10].

Classic KS typically presents as purple, red, or brown-black plaques, macules, or nodules on the face that can spread to the trunk and lower abdomen. These skin lesions are a hallmark of this vascular tumor. In rare cases, classic KS can present without typical skin lesions, making it difficult to diagnose accurately [9]. Classic KS mainly affects men and has a male-to-female ratio of 17:1. It is most common among elderly people of Eastern European and Mediterranean descent, and these individuals have a higher risk of developing secondary cancers [1]. In a retrospective study examining admitted HIV-associated or epidemic KS patients in sub-Saharan Africa, the mortality rate was found to be approximately 20%, with the majority of patients (48%) having an advanced disease with visceral involvement [11].

When considering differential diagnoses, it is important to consider KS in the setting of immune reconstitution inflammatory syndrome (KS-IRIS). This differential is important to consider in a patient with worsening KS after starting ART. Patients may also present with a recurrence of previously treated opportunistic infections, symptoms, or inflammatory processes despite an earlier favorable response to ART [4]. Pulmonary involvement and thrombocytopenia are associated with greater mortality in these patients [4]. Additional symptoms of fever, thrombocytopenia, anemia, hyponatremia, and hypoalbuminemia associated with systemic inflammatory response syndrome (SIRS) suggest a diagnosis of Kaposi sarcoma inflammatory cytokine syndrome (KICS). KICS is also associated with high morbidity and mortality [4]. An excisional lymph node biopsy is important to exclude a diagnosis of multicentric Castleman disease (MCD). MCD may also present with lymphadenopathy, pancytopenia, and SIRS in an HIV patient with Kaposi's sarcoma herpes virus (KSHV) [12]. Additional differential diagnoses include gastrointestinal (GI) malignancies such as adenocarcinoma, which is much more prevalent and accounts for nearly 95% of gastric malignancies in the general population. Other spindle cell tumors that may be appropriate to consider include leiomyomas, rhabdomyosarcomas, pleomorphic sarcomas, and GI stromal tumors [2].

Visceral KS can affect any organ, mainly lymph nodes, lungs, and the GI tract [1]. The most common site of involvement in visceral KS is the GI tract, most commonly the small intestine, large intestine, and stomach [3]. Visceral KS without skin manifestations is rare [13]. For symptomatic patients or individuals with cutaneous disease, endoscopy with biopsy is the most useful method for diagnosing visceral KS [14]. Endoscopically, the appearance of these visceral lesions ranges from flat maculopapular lesions to polypoid masses of varying sizes. Larger lesions have a significant risk of complications within the GI tract, such as hemorrhage, perforation, or obstruction [14]. Endoscopy may be indicated in patients with cutaneous disease to rule out visceral involvement.

KS is a complicated lesion at the microscopic level: in the early stages, there are clusters of uneven spaces lined with endothelial cells surrounding normal skin blood vessels, along with a varying degree of inflammation (referred to as the patch stage). This is followed by the spread of spindle-shaped blood vessels throughout the dermis (the plaque stage), creating channels that hold red blood cells. The later nodular stage of KS is made up of layers of spindle cells, which make up much of the lesion and are believed to be the cancerous component. Most of the spindle cells in KS display markers of endothelial cells, including CD31, CD34, and the vascular endothelial growth factor receptor (VEGFR) [15].

In patch-stage KS lesions, it is common to find sparse chronic inflammatory cells and red blood cells that have leaked out of blood vessels. Kaposi's sarcoma lesions also have a significant presence of macrophages filled with hemosiderin. This is expected, as iron is believed to play a crucial role in the development of KS. Iron staining can be used to differentiate KS from similar-appearing interstitial granuloma annulare lesions, which lack iron [16]. The early histological changes may be subtle and, therefore, may be overlooked during a biopsy. The histology of KS is basically the same regardless of the type of KS. However, some studies have shown minor differences in the histopathology between AIDS-KS and non-HIV-related KS cases. It has been reported that mitosis and cellular anaplasia are more frequent in non-HIV patients, while AIDS-KS lesions tend to have more extensive dissected vessels [16].

The primary goals for the treatment of KS are the alleviation of symptoms and minimizing the progression of the disease. The treatment options for Kaposi sarcoma range from simply monitoring the disease to local treatments to systemic chemotherapy using drugs like paclitaxel and anthracyclines such as doxorubicin and adriamycin. In AIDS-related KS, systemic treatment is centered around combined anti-retroviral therapy (ART), with the need for additional treatment depending on the extent of the disease [10]. Local symptomatic therapy is also a common approach to treatment through intralesional chemotherapy with vinblastine, radiation therapy, and topical alitretinoin for cutaneous KS lesions [17].

In extensive and rapidly progressive KS, both systemic chemotherapy and ART are indicated [18]. Liposomal anthracyclines such as pegylated liposomal doxorubicin and liposomal daunorubicin are the preferred initial systemic chemotherapeutic options for AIDS-related KS. Liposomal anthracyclines have high response rates (30%-60%) and limited risk of cardiotoxicity, allowing for higher doses to be administered if needed [19]. Paclitaxel is another initial chemotherapeutic option for KS, with similar efficacy to liposomal anthracyclines. Although paclitaxel has a more toxic side effect profile than liposomal anthracyclines, their overall response rates (56% to 46%, respectively) and two-year survival rates (79% to 78%, respectively) were nearly identical [20]. Other potential treatment options for advanced KS include etoposide, vinorelbine, gemcitabine, and pomalidomide.

## Conclusions

Our case presents an interesting concurrent presentation of two different pathologies, MAC and visceral KS. Both diagnoses could have individually explained the lymphadenopathy seen in our patient. The enlarging neck mass raised concern for lymphadenitis, possibly related to TB. However, diagnostic investigation allowed the diagnosis of MAC infection. Due to concurrent rectal bleeding in the patient, further investigation and assessment of inguinal lymphadenopathy were pursued. The inguinal lymph node biopsy allowed proper diagnosis while the colonoscopy was initially delayed. Both disease states are important differentials when treating patients with AIDS. A thorough evaluation of HIV lymphadenopathy and knowledge of the significant overlap between these two pathologies is necessary, especially when treating immunocompromised patients.

## References

[1] El Mawla Z, Ghannoum H, Saliba M, Michel Minari A, Kanaan HM (2022). Visceral Kaposi’s sarcoma as a presentation in a newly diagnosed HIV-infected man: a case report. Cureus.

[2] Hauser N, McKenzie D, Fonseca X, Orsini J (2015). Visceral Kaposi’s sarcoma presenting as upper gastrointestinal bleeding. Case Rep Gastrointest Med.

[3] Zeinaty PE, Lebbé C, Delyon J (2023). Endemic Kaposi’s sarcoma. Cancers (Basel).

[4] Poizot-Martin I, Brégigeon S, Palich R (2022). Immune reconstitution inflammatory syndrome associated Kaposi sarcoma. Cancers (Basel).

[5] Falkinham JO (2018). Mycobacterium avium complex: adherence as a way of life. AIMS Microbiol.

[6] Havlik JA Jr, Horsburgh CR Jr, Metchock B, Williams PP, Fann SA, Thompson SE 3rd (1992). Disseminated Mycobacterium avium complex infection: clinical identification and epidemiologic trends. J Infect Dis.

[7] To K, Cao R, Yegiazaryan A, Owens J, Venketaraman V (2020). General overview of nontuberculous mycobacteria opportunistic pathogens: Mycobacterium avium and Mycobacterium abscessus. J Clin Med.

[8] Corti M, Palmero D (2008). Mycobacterium avium complex infection in HIV/AIDS patients. Expert Rev Anti Infect Ther.

[9] Schulberg S, Al-Feghali V, Bain K, Shehebar J (2018). Non-cutaneous AIDS-associated Kaposi's sarcoma presenting as recurrent rectal abscesses. BMJ Case Rep.

[10] Dittmer DP, Damania B (2019). Kaposi’s sarcoma-associated herpesvirus (KSHV)-associated disease in the AIDS patient: an update. Cancer Treat Res.

[11] Vally F, Selvaraj WM, Ngalamika O (2020). Admitted AIDS-associated Kaposi sarcoma patients: Indications for admission and predictors of mortality. Medicine (Baltimore).

[12] Dumic I, Radovanovic M, Igandan O (2020). A fatal case of Kaposi sarcoma immune reconstitution syndrome (KS-Iris) complicated by Kaposi sarcoma inflammatory cytokine syndrome (KICS) or multicentric Castleman disease (MCD): a case report and review. Am J Case Rep.

[13] Tesfaye B, Dagne R, Brown K, Horwitz SL (2015). AIDS-related visceral Kaposi sarcoma (KS) presenting with protein-losing enteropathy (PLE). Am J Gastroenterol.

[14] Nagata N, Shimbo T, Yazaki H (2012). Predictive clinical factors in the diagnosis of gastrointestinal Kaposi's sarcoma and its endoscopic severity. PLoS One.

[15] Boshoff C (2002). Kaposi's sarcoma biology. IUBMB Life.

[16] Radu O, Pantanowitz L (2013). Kaposi sarcoma. Arch Pathol Lab Med.

[17] Donato V, Guarnaccia R, Dognini J, de Pascalis G, Caruso C, Bellagamba R, Morrone A (2013). Radiation therapy in the treatment of HIV-related Kaposi's sarcoma. Anticancer Res.

[18] Bower M, Palfreeman A, Alfa-Wali M (2014). British HIV Association guidelines for HIV-associated malignancies 2014. HIV Med.

[19] Petre CE, Dittmer DP (2007). Liposomal daunorubicin as treatment for Kaposi's sarcoma. Int J Nanomedicine.

[20] Cianfrocca M, Lee S, Von Roenn J (2010). Randomized trial of paclitaxel versus pegylated liposomal doxorubicin for advanced human immunodeficiency virus-associated Kaposi sarcoma: evidence of symptom palliation from chemotherapy. Cancer.

